# The genome sequence of the black arches, *Lymantria monacha *(Linnaeus, 1758)

**DOI:** 10.12688/wellcomeopenres.17817.1

**Published:** 2022-04-05

**Authors:** Douglas Boyes, University of Oxford and Wytham Woods Genome Acquisition Lab, Darwin Tree of Life Barcoding collective, Wellcome Sanger Institute Tree of Life programme, Wellcome Sanger Institute Scientific Operations: DNA Pipelines collective, Tree of Life Core Informatics collective, Dominic Phillips, Darwin Tree of Life Consortium

**Affiliations:** 1 UK Centre for Ecology and Hydrology, Wallingford, Oxfordshire, UK; 2 Department of Life Science, Natural History Museum, London, UK

**Keywords:** Lymantria monacha, black arches, genome sequence, chromosomal, Lepidoptera

## Abstract

We present a genome assembly from an individual male *Lymantria monacha* (the black arches; Arthropoda; Insecta; Lepidoptera; Erebidae). The genome sequence is 916 megabases in span. The majority of the assembly (99.99%) is scaffolded into 28 chromosomal pseudomolecules, with the Z sex chromosome assembled. The mitochondrial genome was also assembled, and is 15.6 kilobases in length.

## Species taxonomy

Eukaryota; Metazoa; Ecdysozoa; Arthropoda; Hexapoda; Insecta; Pterygota; Neoptera; Endopterygota; Lepidoptera; Glossata; Ditrysia; Noctuoidea; Erebidae; Lymantriinae; Lymantria; *Lymantria monacha* (Linnaeus, 1758) (NCBI:txid988002).

## Background

*Lymantria monacha* (black arches) is a nocturnal, univoltine species typically identified by the numerous black markings across its white forewing. Though melanic forms can be found, these are identified by the shared pink bands present on the abdomen of each form. The larvae of *L.monacha* mainly feed on species in the *Quercus* genus, but have been recorded on a wide range of host plant species. Though local to the United Kingdom, *L.monacha* also occurs throughout the Palearctic region and east to India and Japan. Notably, *L.monacha* is a pest species of pine and spruce as well as being an invasive timber pest in North America ( Keena, 2003; Prestemon *et al*., 2008). Climate change has shown an increase in *L.monacha* distribution in Northern Europe and due to the destruction it causes to affected species, this can lead to an increase in secondary pests and infections ( Fält-Nardmann *et al*., 2018; Økland *et al*., 2019). As such, providing a full genome of this species may contribute to the varied methods being developed to control this species.

The genome of *L.monacha* was sequenced as part of the Darwin Tree of Life Project, a collaborative effort to sequence all of the named eukaryotic species in the Atlantic Archipelago of Britain and Ireland. Here we present a chromosomally complete genome sequence for *L.monacha* , based on one specimen from Wytham Woods, Oxfordshire, UK.

## Genome sequence report

**Figure 1.**
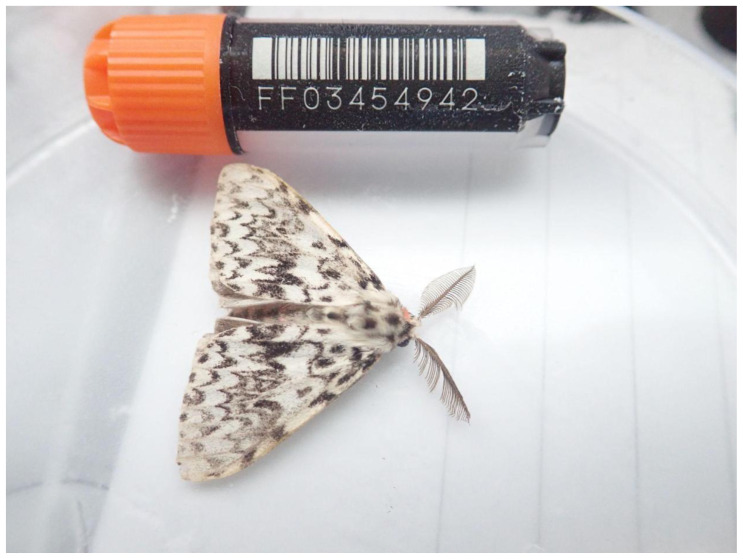
Image of the *Lymantria monacha* (ilLymMona1) specimen taken prior to preservation and processing. Specimen shown next to FluidX storage tube, 43.9 mm in length.

The genome was sequenced from one male *L. monacha* ( Figure 1) collected from Wytham Woods, Oxfordshire (biological vice-county: Berkshire), UK (latitude 51.764, longitude -1.327). A total of 39-fold coverage in Pacific Biosciences single-molecule long reads and 52-fold coverage in 10X Genomics read clouds were generated. Primary assembly contigs were scaffolded with chromosome conformation Hi-C data. Manual assembly curation corrected 33 missing/misjoins and removed 12 haplotypic duplications, reducing the assembly size by 0.20% and the scaffold number by 28.57%, and increasing the scaffold N50 by 1.14%.

**Figure 2.**
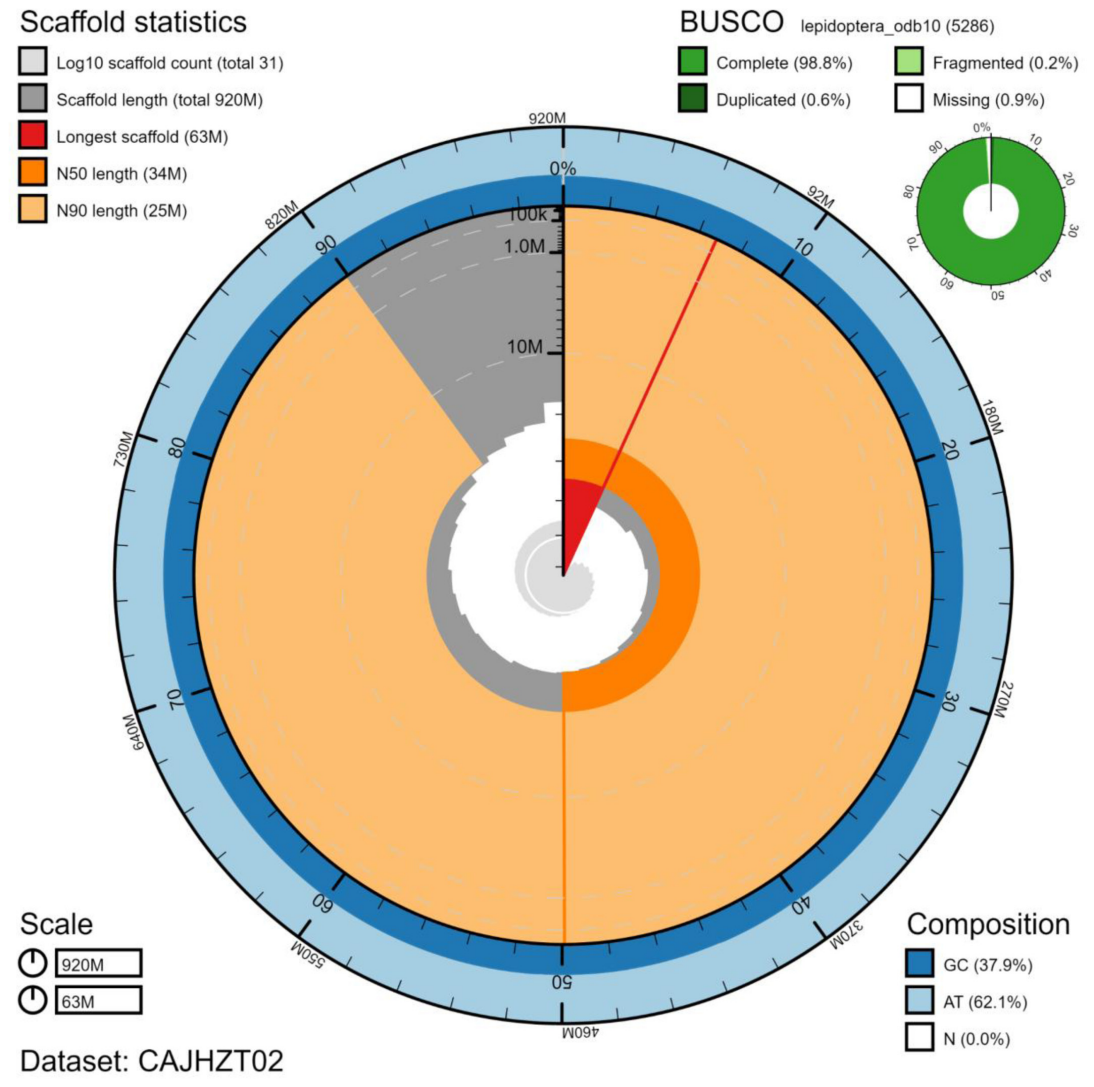
Genome assembly of *Lymantria monacha*, ilLymMona1.2: metrics. The BlobToolKit Snailplot shows N50 metrics and BUSCO gene completeness. The main plot is divided into 1,000 size-ordered bins around the circumference with each bin representing 0.1% of the 915,668,749 bp assembly. The distribution of scaffold lengths is shown in dark grey with the plot radius scaled to the longest scaffold present in the assembly (62,811,790 bp, shown in red). Orange and pale-orange arcs show the N50 and N90 scaffold lengths (33,870,686 and 24,894,941 bp), respectively. The pale grey spiral shows the cumulative scaffold count on a log scale with white scale lines showing successive orders of magnitude. The blue and pale-blue area around the outside of the plot shows the distribution of GC, AT and N percentages in the same bins as the inner plot. A summary of complete, fragmented, duplicated and missing BUSCO genes in the lepidoptera_odb10 set is shown in the top right. An interactive version of this figure is available at https://blobtoolkit.genomehubs.org/view/ilLymMona1.2/dataset/CAJHZT02/snail.

The final assembly has a total length of 916 Mb in 30 sequence scaffolds with a scaffold N50 of 12.7 Mb ( Table 1). The majority of the assembly sequence (99.99%) was assigned to 28 chromosomal-level scaffolds, representing 27 autosomes (numbered by sequence length), and the Z sex chromosome ( Figure 2– Figure 5; Table 2). The assembly has a BUSCO v5.2.2 ( Manni *et al*., 2021) completeness of 98.8% (single 98.2%, duplicated 0.6%) using the lepidoptera_odb10 reference set. While not fully phased, the assembly deposited is of one haplotype. Contigs corresponding to the second haplotype have also been deposited.

**Table 1.** Genome data for *Lymantria monacha*, ilLymMona1.2.

*Project accession data*	
Assembly identifier	ilLymMona1.2
Species	*Lymantria monacha*
Specimen	ilLymMona1
NCBI taxonomy ID	NCBI:txid78897
BioProject	PRJEB42133
BioSample ID	SAMEA7519912
Isolate information	Male, thorax/abdomen (genome assembly), head (Hi-C)
*Raw data accessions*	
PacificBiosciences SEQUEL II	ERR6576315-ERR6576316
10X Genomics Illumina	ERR6002667-ERR6002674
Hi-C Illumina	ERR6002675-ERR6002677
PolyA RNA-Seq Illumina	ERR6286706
*Genome assembly*	
Assembly accession	GCA_905163515.2
Accession of alternate haplotype	GCA_905163525.1
Span (Mb)	916
Number of contigs	47
Contig N50 length (Mb)	33.5
Number of scaffolds	30
Scaffold N50 length (Mb)	33.9
Longest scaffold (Mb)	39.6
BUSCO * genome score	C:98.8%[S:98.2%,D:0.6%],F:0.2%,M:0.9%,n:5286

*BUSCO scores based on the lepidoptera_odb10 BUSCO set using v5.2.2. C= complete [S= single copy, D=duplicated], F=fragmented, M=missing, n=number of orthologues in comparison. A full set of BUSCO scores is available at https://blobtoolkit.genomehubs.org/view/ilLymMona1.2/dataset/CAJHZT02/busco.

**Figure 3.**
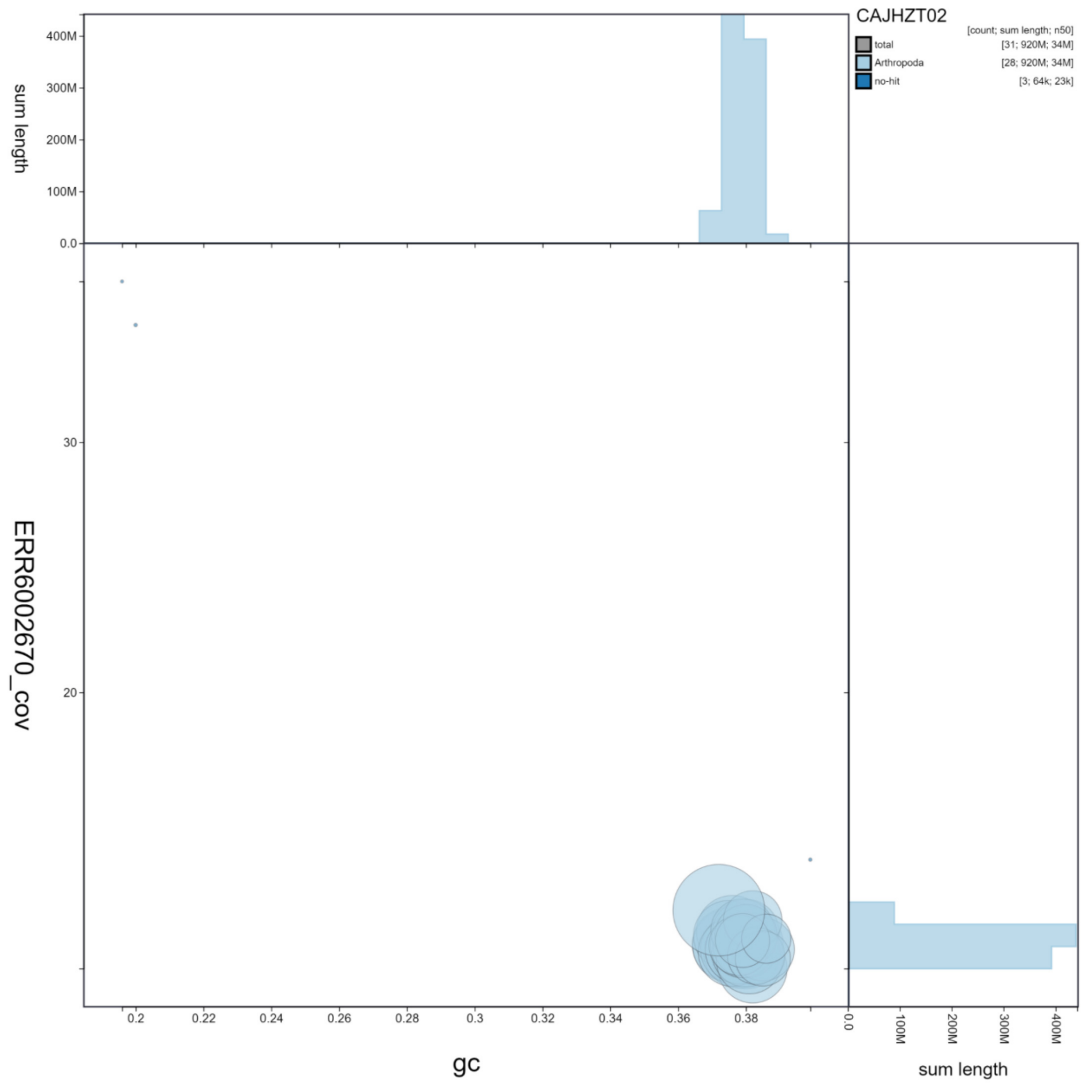
Genome assembly of *Lymantria monacha*, ilLymMona1.2: GC coverage. BlobToolKit GC-coverage plot. Scaffolds are coloured by phylum. Circles are sized in proportion to scaffold length Histograms show the distribution of scaffold length sum along each axis. An interactive version of this figure is available at https://blobtoolkit.genomehubs.org/view/ilLymMona1.2/dataset/CAJHZT02/blob.

**Figure 4.**
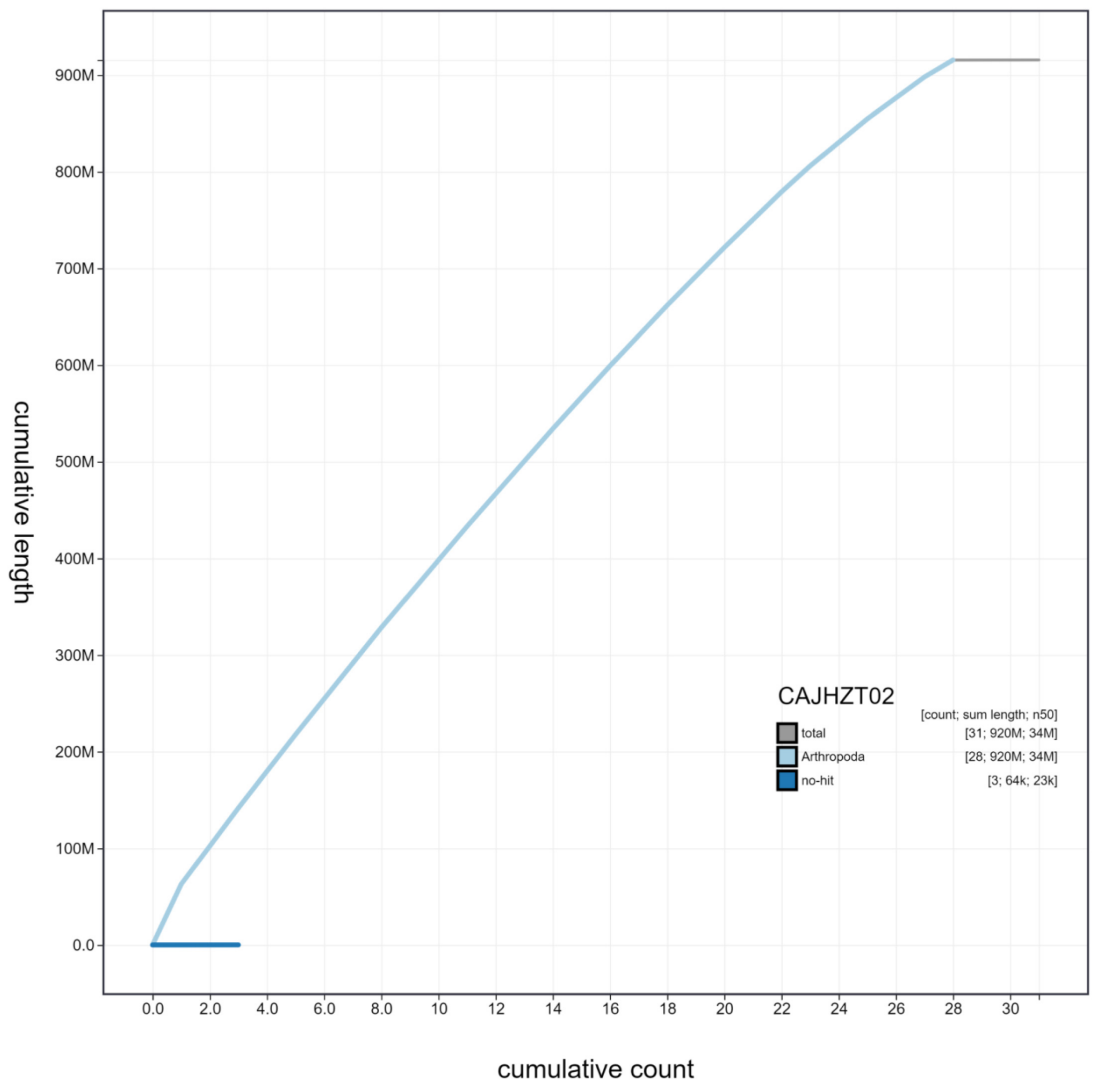
Genome assembly of *Lymantria monacha*, ilLymMona1.2: cumulative sequence. BlobToolKit cumulative sequence plot. The grey line shows cumulative length for all scaffolds. Coloured lines show cumulative lengths of scaffolds assigned to each phylum using the buscogenes taxrule. An interactive version of this figure is available at https://blobtoolkit.genomehubs.org/view/ilLymMona1.2/dataset/CAJHZT02/cumulative.

**Figure 5.**
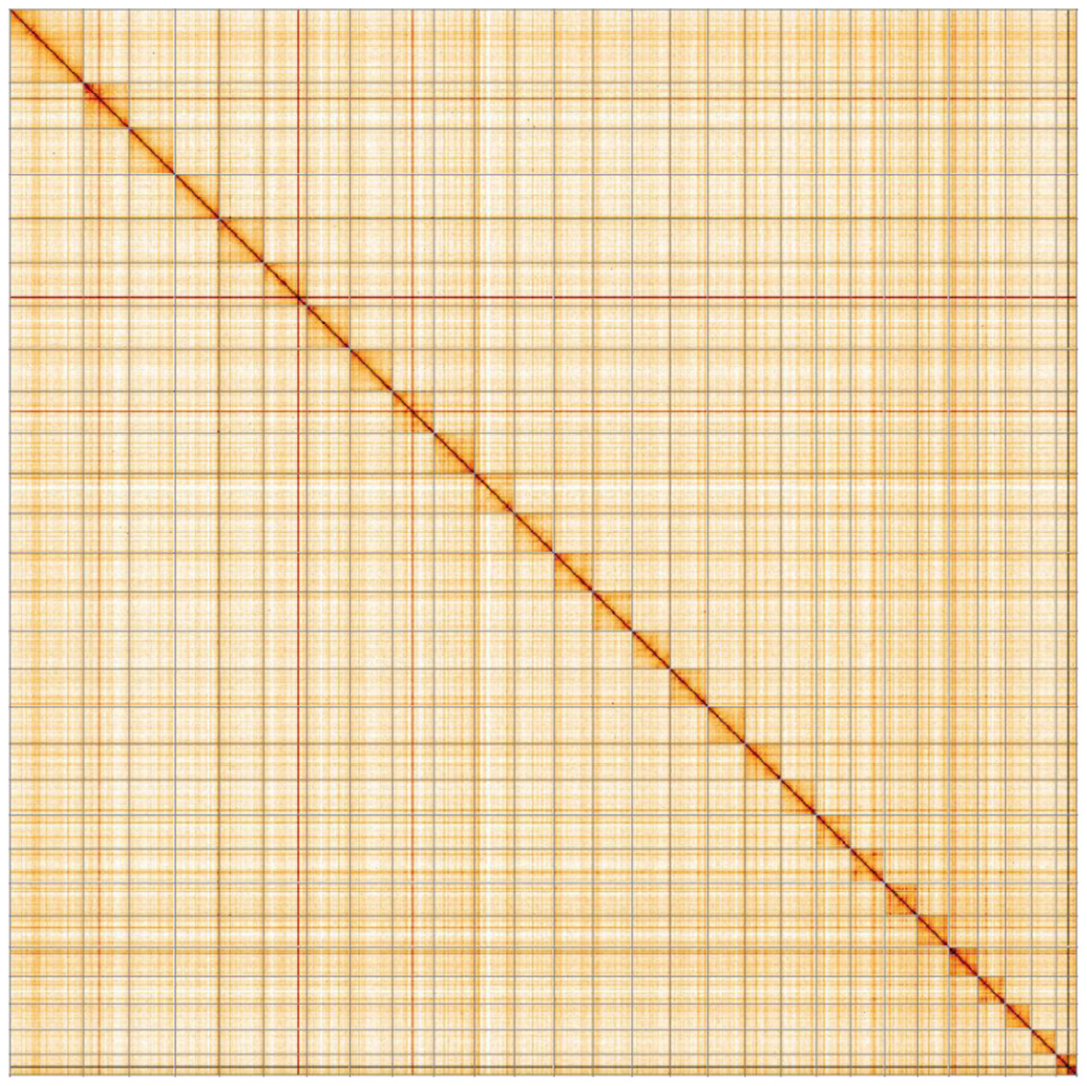
Genome assembly of *Lymantria monacha*, ilLymMona1.2: Hi-C contact map. Hi-C contact map of the ilLymMona1.2 assembly, visualised in HiGlass. Chromosomes are shown in order of size from left to right and top to bottom. An interactive version of this map is available here.

**Table 2.** Chromosomal pseudomolecules in the genome assembly of *Lymantria monacha*, ilLymMona1.2.

INSDC accession	Chromosome	Size (Mb)	GC%
LR991082.1	1	39.55	37.5
LR991083.1	2	39.47	37.5
LR991084.1	3	38.03	37.6
LR991085.1	4	37.87	37.9
LR991086.1	5	37.11	37.5
LR991087.1	6	37.07	37.8
LR991088.1	7	36.45	37.7
LR991089.1	8	35.57	38.0
LR991090.1	9	34.84	38.2
LR991091.1	10	34.35	37.6
LR991092.1	11	33.87	37.8
LR991093.1	12	33.65	38.1
LR991094.1	13	33.49	38.0
LR991095.1	14	32.64	38.0
LR991096.1	15	32.37	38.0
LR991097.1	16	31.88	38.1
LR991098.1	17	30.87	38.0
LR991099.1	18	30.36	37.9
LR991100.1	19	29.48	37.9
LR991101.1	20	29.15	38.5
LR991102.1	21	28.34	38.0
LR991103.1	22	26.80	37.8
LR991104.1	23	24.89	38.2
LR991105.1	24	23.80	38.3
LR991106.1	25	22.12	38.5
LR991107.1	26	21.25	37.9
LR991108.1	27	17.51	38.6
LR991081.1	Z	62.81	37.2
LR991109.2	MT	0.02	19.7
-	Unplaced	0.05	29.3

## Methods

### Sample acquisition and nucleic acid extraction

A single male *L. monacha* (ilLymMona1) was collected from Wytham Woods, Oxfordshire (biological vice-county: Berkshire), UK (latitude 51.772, longitude -1.338) by Douglas Boyes, UKCEH, using a light trap in woodland. The sample was identified by the same individual, and preserved on dry ice.

DNA was extracted from thorax/abdomen tissue of ilLymMona1 at the Wellcome Sanger Institute (WSI) Scientific Operations core from the whole organism using the Qiagen MagAttract HMW DNA kit, according to the manufacturer’s instructions. RNA was extracted from remaining thorax/abdomen tissue of ilLymMona1 in the Tree of Life Laboratory at the WSI using TRIzol, according to the manufacturer’s instructions. RNA was then eluted in 50 μl RNAse-free water and its concentration RNA assessed using a Nanodrop spectrophotometer and Qubit Fluorometer using the Qubit RNA Broad-Range (BR) Assay kit. Analysis of the integrity of the RNA was done using Agilent RNA 6000 Pico Kit and Eukaryotic Total RNA assay. 

### Sequencing

Pacific Biosciences HiFi circular consensus and 10X Genomics Chromium read cloud sequencing libraries were constructed according to the manufacturers’ instructions. Poly(A) RNA-Seq libraries were constructed using the NEB Ultra II RNA Library Prep kit. Sequencing was performed by the Scientific Operations core at the Wellcome Sanger Institute on Pacific Biosciences SEQUEL II (HiFi), Illumina HiSeq X (10X) and Illumina HiSeq 4000 (RNA-Seq) instruments. Hi-C data were generated from head tissue of ilLymMona1 using the Qiagen EpiTect Hi-C kit and sequenced on a HiSeq X instrument.

### Genome assembly

Assembly was carried out with Hifiasm ( Cheng *et al*., 2021); haplotypic duplication was identified and removed with purge_dups ( Guan *et al*., 2020) without the -e flag. One round of polishing was performed by aligning 10X Genomics read data to the assembly with longranger align, calling variants with freebayes ( Garrison & Marth, 2012). The assembly was then scaffolded with Hi-C data ( Rao *et al*., 2014) using SALSA2 ( Ghurye *et al*., 2019). The assembly was checked for contamination and corrected using gEVAL ( Chow *et al*., 2016) as described previously ( Howe *et al*., 2021). Manual curation ( Howe *et al*., 2021) was performed using gEVAL, HiGlass ( Kerpedjiev *et al*., 2018) and Pretext. The mitochondrial genome was assembled using MitoHiFi ( Uliano-Silva *et al*., 2021), which performs annotation using MitoFinder ( Allio *et al*., 2020). The genome was analysed and BUSCO scores generated within the BlobToolKit environment ( Challis *et al*., 2020). Table 3 contains a list of all software tool versions used, where appropriate.

**Table 3.** Software tools used.

Software tool	Version	Source
Hifiasm	0.12	Cheng *et al*., 2021
purge_dups	1.2.3	Guan *et al*., 2020
SALSA	2.2	Ghurye *et al*., 2019
longranger align	2.2.2	https://support.10xgenomics.com/genome-exome/software/pipelines/latest/advanced/other-pipelines
freebayes	1.3.1-17-gaa2ace8	Garrison & Marth, 2012
MitoHiFi	1.0	Uliano-Silva *et al*., 2021
gEVAL	N/A	Chow *et al*., 2016
HiGlass	1.11.6	Kerpedjiev *et al*., 2018
PretextView	0.1.x	https://github.com/wtsi-hpag/PretextView
BlobToolKit	3.0.5	Challis *et al*., 2020

### Ethics/compliance issues

The materials that have contributed to this genome note have been supplied by a Darwin Tree of Life Partner. The submission of materials by a Darwin Tree of Life Partner is subject to the Darwin Tree of Life Project Sampling Code of Practice. By agreeing with and signing up to the Sampling Code of Practice, the Darwin Tree of Life Partner agrees they will meet the legal and ethical requirements and standards set out within this document in respect of all samples acquired for, and supplied to, the Darwin Tree of Life Project. Each transfer of samples is further undertaken according to a Research Collaboration Agreement or Material Transfer Agreement entered into by the Darwin Tree of Life Partner, Genome Research Limited (operating as the Wellcome Sanger Institute), and in some circumstances other Darwin Tree of Life collaborators.

## Data availability

European Nucleotide Archive: Lymantria monacha (black arches). Accession number PRJEB42133; https://identifiers.org/ena.embl/PRJEB42133.

The genome sequence is released openly for reuse. The *L. monacha* genome sequencing initiative is part of the Darwin Tree of Life (DToL) project. All raw sequence data and the assembly have been deposited in INSDC databases. The genome will be annotated using the RNA-Seq data and presented through the Ensembl pipeline at the European Bioinformatics Institute. Raw data and assembly accession identifiers are reported in Table 1.

## Author information

Members of the University of Oxford and Wytham Woods Genome Acquisition Lab are listed here: https://doi.org/10.5281/zenodo.5746938.

Members of the Darwin Tree of Life Barcoding collective are listed here: https://doi.org/10.5281/zenodo.5744972.

Members of the Wellcome Sanger Institute Tree of Life programme are listed here: https://doi.org/10.5281/zenodo.6125027.

Members of Wellcome Sanger Institute Scientific Operations: DNA Pipelines collective are listed here: https://doi.org/10.5281/zenodo.5746904.

Members of the Tree of Life Core Informatics collective are listed here: https://doi.org/10.5281/zenodo.6125046.

Members of the Darwin Tree of Life Consortium are listed here: https://doi.org/10.5281/zenodo.5638618.
