# Supplementary material for: The genome sequence of the black arches, Lymantria monacha (Linnaeus, 1758)
Source: Wellcome Open Res. 2022 Apr 5;7:128. [Version 1] doi: 10.12688/wellcomeopenres.17817.1 (PMC10622856; doi:10.12688/wellcomeopenres.17817.1)
Supplement: Peer review history

# Reviewer report: Reviewer response for version 1

**DOI:** 10.21956/wellcomeopenres.19726.r68797

**Published:** 2023-11-02

**Authors:** Themistoklis Giannoulis

**Affiliations:** 1 Laboratory of Genetics, Faculty of Animal Science, University of Thessaly, Larissa, Greece

The Manuscript is very targeted and is a part of a large effort, The Tree of Life, which is a fascinating work which will provide reference genomes for a variety of species. Towards this, the manuscript fulfils its purpose and provides a genome for the black arches, which could be used for the development of methods to control the expansion of the species and limit its destructive actions. The only thing I want to mention is what was the purpose of extracting RNA? Since it was not used in the experiment described downstream.

Are sufficient details of methods and materials provided to allow replication by others?

Yes

Is the rationale for creating the dataset(s) clearly described?

Yes

Are the datasets clearly presented in a useable and accessible format?

Yes

Are the protocols appropriate and is the work technically sound?

Yes

Reviewer Expertise:

Genetics, animal genomics, bioinformatics

I confirm that I have read this submission and believe that I have an appropriate level of expertise to confirm that it is of an acceptable scientific standard.

# Reviewer report: Reviewer response for version 1

**DOI:** 10.21956/wellcomeopenres.19726.r50048

**Published:** 2022-05-09

**Authors:** Mathilde Cordellier

**Affiliations:** 1 Institute for Zoology, Universität Hamburg, Hamburg, Germany

This study presents the fully assembled chromosome level genome of the black arches, a moth with a wide palearctic distribution.

 I find this genome report to be well written and clear, with the adequate amount of information provided. The sequencing effort resulted in an amazing assembly I am sure will be helpful in the future, and I only have minor comments. I was very shocked and sad to discover the first author has passed away, and hope these questions can be addressed, nevertheless.

 “Increase in distribution”: do the authors mean an increase in frequency/occurrence?

 Is there a reason why a male was sequenced and not a female? The assembly is then missing the W chromosome.

 From my experience, it is difficult to obtain assemblies without contamination. Although thorax/abdomen tissue were used, there is virtually no assembled sequence from other organisms. I am wondering how this was possible. Were measures taken to avoid contamination through gut content, ectoparasites, surface and internal bacteria? I do trust the assembly, my question is merely aimed at adding valuable information for others, whose work would greatly benefit from a protocol reducing contamination!

 Would it be possible to number the chromosomes on the HiC contact map, to establish a correspondence between the figure and Table 2?

Are sufficient details of methods and materials provided to allow replication by others?

Yes

Is the rationale for creating the dataset(s) clearly described?

Yes

Are the datasets clearly presented in a useable and accessible format?

Yes

Are the protocols appropriate and is the work technically sound?

Yes

Reviewer Expertise:

population genomics, freshwater ecology, arthropod genome evolution, evolutionary biology

I confirm that I have read this submission and believe that I have an appropriate level of expertise to confirm that it is of an acceptable scientific standard.
